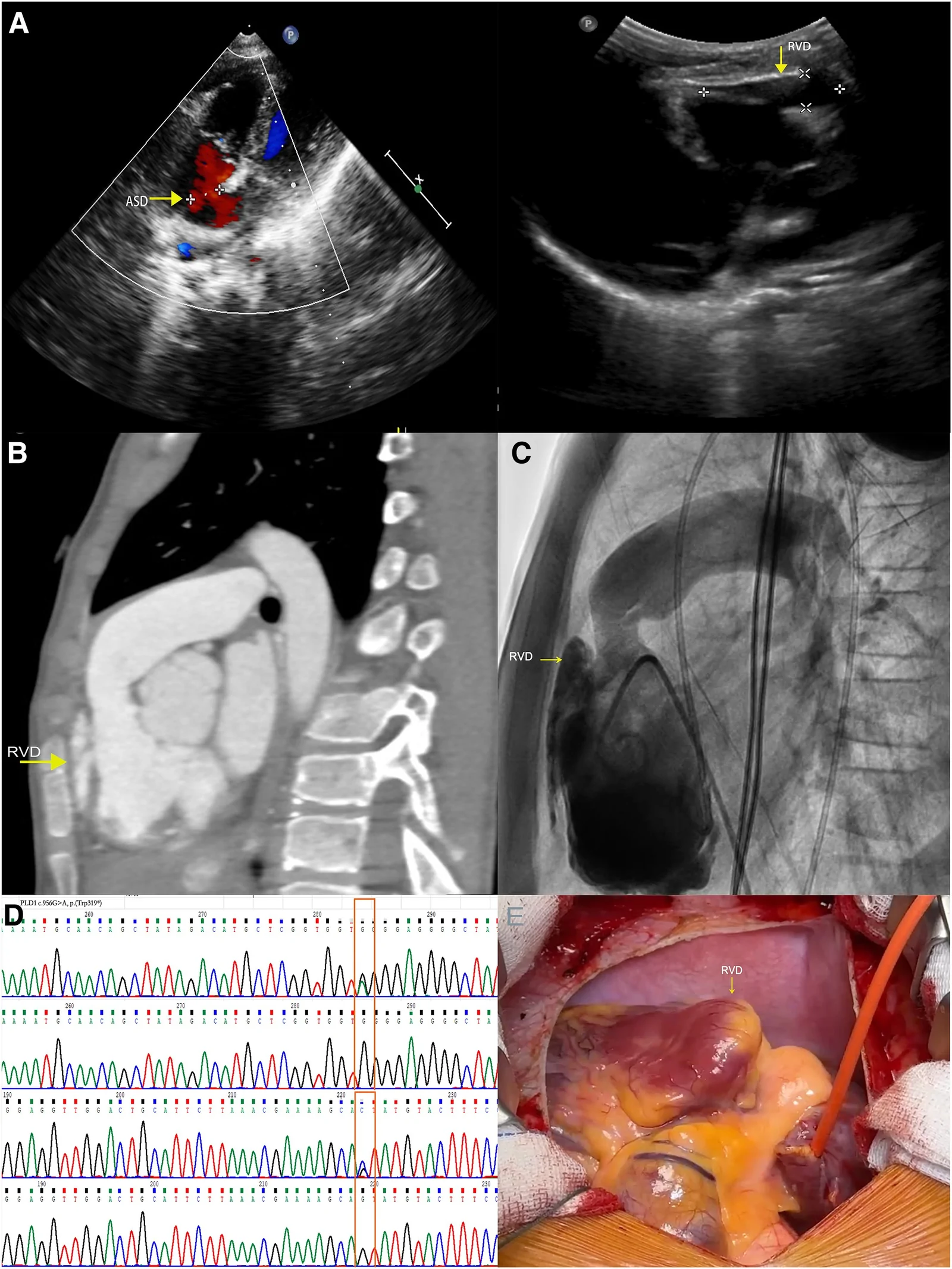# A case report of right ventricular cardiomyopathy with right ventricular diverticulum and atrial septal defect associated with biallelic PLD1 gene variants

**DOI:** 10.1093/ehjcr/ytag688

**Published:** 2026-09-15

**Authors:** Yong Liu, Meng Zhang, Tingliang Liu

**Affiliations:** Department of Pediatrics, Xiamen Chang Gung Hospital, Huaqiao University, 123 Xiafei Road, Haicang, Xiamen, Fujian 361028, China; Department of Cardiovascular, Shanghai Children's Medical Center, School of Medicine, Shanghai Jiao Tong University, 1678 Dongfang Road, Pudong New Area, Shanghai 200127, China; Department of Cardiovascular, Shanghai Children's Medical Center, School of Medicine, Shanghai Jiao Tong University, 1678 Dongfang Road, Pudong New Area, Shanghai 200127, China; Department of Cardiovascular, Shanghai Children's Medical Center, School of Medicine, Shanghai Jiao Tong University, 1678 Dongfang Road, Pudong New Area, Shanghai 200127, China

A 12-year-old female patient presented with a history of cyanosis and reduced exercise tolerance for over 10 years, with one episode of syncope. Physical examination revealed cyanosis of the peripheral skin and nail beds of the extremities, clubbing of the fingers and toes, and a blood oxygen saturation of 86%–88%. Electrocardiography revealed non-paroxysmal atrioventricular junctional tachycardia, and IRBBB pattern. Transthoracic echocardiography demonstrated an atrial septal defect with bidirectional shunt (predominantly right-to-left), and diverticulum-like change in the anterior wall of the right ventricle with reduced right ventricular diastolic function (*Panel A*). Cardiac magnetic resonance imaging and cardiac computed tomography angiography revealed the right ventricular anterior wall aneurysm measuring ∼3.4 cm × 1.1 cm × 3.3 cm, a ventricular septum protruding into the left ventricle, left ventricular myocardial laxity, and subendocardial delayed enhancement in the right ventricle (*Panel B*). Cardiac catheterization revealed normal pulmonary artery pressure: right ventricular pressure 18/6 mmHg, pulmonary artery pressure 16/10/13 mmHg, mean pulmonary artery pressure 13 mmHg (<25 mmHg), and normal pulmonary vascular resistance (*Panel C*). Genetic testing revealed heterozygous variants in the PLD1 gene: a novel nonsense variant (c.956G > A, p.Trp319*) and a splice-site variant (c.1227 + 1G > C). According to the ACMG/AMP guidelines, the c.956G > A (p.Trp319*) variant is classified as Pathogenic (PVS1, PM2, PP3), and the c.1227 + 1G > C variant is classified as Pathogenic (PVS1, PM2, PP3) (*Panel D*). During the surgical procedure, the cardiac diverticulum was removed. The intraoperative presentation is shown in *Panel E*. Postoperatively, the cardiac magnetic resonance imaging (MRI) of the child showed that the right ventricular function had recovered well and the blood oxygen saturation had returned to normal. No recurrence of hypoxaemia was observed during the 6-month postoperative follow-up period.

The PLD1 gene is located at 3q26.31 and is widely expressed during the early stages of cardiac development. The protein it encodes catalyses the hydrolysis of phosphatidylcholine into phosphoric acid and choline, and is involved in various cellular biological pathways, such as signal transduction, membrane transport and the regulation of mitosis.^1,2^ In this case, a PLD1 mutation may led to a series of malformations: a right ventricular aneurysm, right ventricular dysfunction and an atrial septal defect, constituting an extremely rare congenital malformation. Multimodal imaging studies [echocardiography, computed tomography (CT), MRI, and cardiac catheterization] played a crucial role in the diagnosis, whilst genetic testing revealed the genetic aetiology, providing a basis for precise diagnosis and treatment. The patient successfully underwent right ventricular aneurysm resection and repair, as well as atrial septal defect repair. The mechanism of the right-to-left shunt is driven by restrictive right ventricular physiology, the combined effect of the right ventricle (RV) diastolic dysfunction, outflow tract obstruction, and the haemodynamic ‘dead space’ effect of the RV diverticulum results in elevated right atrial pressure, driving the right-to-left shunt across the atrial septal defect (ASD). This also explains the leftward deviation of the interventricular septum. The surgery simultaneously corrected the shunt and right ventricular structural abnormalities, improving the patient’s haemodynamic status. Postoperative recovery was uneventful, with no complications. This case expands the phenotypic spectrum of PLD1-associated congenital heart disease and illustrates that right-to-left interatrial shunting can occur through restrictive RV physiology in the absence of pulmonary hypertension. While surgical repair of the structural defects effectively corrects the haemodynamic compromise, the underlying PLD1-related cardiomyopathy is irreversible, mandating lifelong structured cardiac surveillance.

The patient's legal guardian provided written informed consent for the publication of their anonymized case details and images.

**Figure:**